# Five-year survival rate of kidney cancer (localized renal cell carcinoma) in the Asia: A systematic review and meta-analysis

**DOI:** 10.1097/MD.0000000000043867

**Published:** 2025-08-22

**Authors:** Hamed Delam, Reza Zare, Zahra Keshtkaran, Ahmadreza Eidi, Mohammad Sadegh Moradi Sarcheshmeh, Alireza Shahedi, Soheil Hassanipour, Meghdad Abdollahpour-Alitappeh, Ehsan Amini-Salehi

**Affiliations:** a Student Research Committee, Larestan University of Medical Sciences, Larestan, Iran; b Department of Epidemiology, Student Research Committee, Shiraz University of Medical Sciences, Shiraz, Iran; c Department of Nursing, Community Based Psychiatric Care Research Center, School of Nursing and Midwifery, Shiraz University of Medical Sciences, Shiraz, Iran; d Emergency Medical System, Larestan University of Medical Sciences, Larestan, Iran; e Bachelor of Surgical Technology, Nurse and Midwifery Faculty, Shahed University, Tehran, Iran; f Students’ Scientific Research Center (SSRC), Tehran University of Medical Sciences, Tehran, Iran; g Gastrointestinal and Liver Diseases Research Center, Guilan University of Medical Sciences, Rasht, Iran; h Department of Physiology and Pharmacology, Pasteur Institute of Iran, Tehran, Iran.

**Keywords:** Asia, kidney neoplasms, renal cell carcinoma, survival rate

## Abstract

**Background:**

The highest incidence and mortality rates of this cancer have been observed in Asia. In the current research, an estimate of localized renal cell carcinoma survival in Asia is presented.

**Methods:**

In the present meta-analysis study, 6 scientific databases of the world were used to search for related articles. Inclusion criteria included cross-sectional, cohort and case-control studies. Articles published until the middle of 2022 were reviewed. The risk of bias in the studies was reduced by using the random-effects model.

**Results:**

Overall, 41 papers had criteria for addition in the analysis. Survival in 5 years was at 68.60% (95% CI 65.80–71.30). The meta-regression results displayed an important association among publish year and survival rate. Also, human development index was a reason of variability in finding of survival rate.

**Conclusion:**

Compared to European countries and the United States, the survival rate for kidney cancer in Asia is lower.

## 1. Introduction

Chronic illnesses are currently a major public health concern worldwide.^[1]^ Kidney cancer is a common malignancy with an unfavorable prognosis.^[2]^ This cancer is account for 2% to 3% of all cancers.^[1,3,4]^ In 2018, kidney cancer was diagnosed as the 15th most common cancer.^[5]^ Between the different areas, Asia had the uppermost incidence rate and death rate. (36% of all cases and 45% of deaths), and Oceania also had the lowest incidence and mortality rates (1.2% of all cases and 0.9% of deaths).^[5]^

Although kidney cancer is rare compared to other cancers, it is still increasing in incidence. However, the mortality rate has stayed constant in both developed and developing countries. The leading cause of kidney cancer is still unknown, but the risk factors for smoking,^[6]^ obesity and hypertension,^[7]^ chemical carcinogens,^[8,9]^ diuretics,^[10,11]^ and other factors such as occupational factors, history of kidney stones, diet and drinks, reproductive factors, hormones, and genetics can also play a role in the risk of kidney cancer.^[12,13]^

Survival statistics are the most widely used measure of the prognosis of cancer patients and their possible disease progression; hence it is of interest to patients, physicians, researchers, and policymakers. Although a seemingly simple concept, survival can be confusing, and the overall survival rate is the number of survivors among people with cancer.^[14]^

So far, many studies have been conducted regarding the survival rate of kidney cancer patients in different years and at different times, and each of them has mentioned different numbers, and contradictions can be seen between the results in different studies. On the one hand, no systematic review and meta-analysis study has been designed to examine and integrate these contradictions in Asia, so the results of this study provide a good basis for health-treatment planning and knowledge of the epidemiological situation, especially the survival rate of kidney cancer for health policy makers.

## 2. Methods

### 2.1. Research plan

This study examines the survival rates of localized renal cell carcinoma (RCC) in Asian countries through systematic review and meta-analysis. An electronic search of international scientific databases was done. The design of this study was done in 2022. The method was based on PRISMA, which is the preferred reporting items for systematic reviews and meta-analysis.^[15]^

### 2.2. Search approach

The study’s authors searched 6 international databases, including PubMed/Medline, EMBASE, Scopus, Google Scholar, ISI (Web of Knowledge), and ProQuest, until June 1, 2022. Selected keywords for international databases included: (“Kidney Cancer,” “kidney Neoplasms,” “kidney carcinoma,” “Cancer of kidney,” “ Neoplasms of kidney,” “localized RCC “, “RCC,” “Survival,” “Survival Analysis,” “Survival Rate,” “Asian countries,” “Afghanistan,” “Armenia,” “Azerbaijan,” “Bahrain,” “ Bangladesh,” “Bhutan,” “Brunei,” “Myanmar,” “Cambodia,” “China,” “Georgia,” “Hong Kong,” “India,” “Indonesia,” “Iran,” “Iraq,” “Japan,” “Jordan,” “Kazakhstan,” “North Korea,” “South Korea,” “Kuwait,” “Kyrgyzstan,” “Laos,” “Lebanon,” “Macau,” “Malaysia,” “Maldives,” “ Mongolia,” “Nepal,” “Oman,” “Pakistan,” “Philippines,” “Qatar,” “Saudi Arabia,” “Singapore,” “Sri Lanka,” “Syria,” “Taiwan,” “ Tajikistan,” “Thailand,” “Timor-Leste,” “Turkmenistan,” “United Arab Emirates,” “Uzbekistan,” “Vietnam,” and “Yemen”) (Supplementary 1, Supplemental Digital Content, https://links.lww.com/MD/P661).

EndNote X8 software was used to input the collected information for document review and management, and duplicate articles were automatically deleted. Two researchers independently extracted and reviewed the information necessary for the articles after electronically searching the scientific databases. Any potential disagreements were resolved by a third researcher.

### 2.3. Inclusion criteria

The list contained all observational studies (cross-sectional, case-control, and cohort studies) that were published up until June 1, 2022. The study only included survival and RCC that were exclusively localized in Asian countries. Articles were not limited in time or language.

### 2.4. Exclusion criteria

Studies that evaluated other types of cancer, review studies and meta-analyses, letters to the editor, studies that included specific treatments (clinical trials), cellular, laboratory, and animal articles were not reviewed. The present study did not include a gray literature search, which included summaries of conferences, seminars, and statistics provided by organizations.

### 2.5. Quality assessment

The quality of articles is being evaluated using a checklist called The Newcastle-Ottawa Quality Assessment Form. This tool has 3 different parts including selection (4 questions), comparability (1 question) and outcome (3 questions) and based on the final scores divided into 3 categories: good (3 or 4 stars in selection domain and 1 or 2 stars in comparability domain and 2 or 3 stars in outcome/exposure domain), fair (2 stars in selection domain and 1 or 2 stars in comparability domain and 2 or 3 stars in outcome/exposure domain) and poor (0 or 1 star in selection domain or 0 stars in comparability domain or 0 or 1 stars in outcome/exposure domain).^[16]^ The Strobe checklist’s content analysis was used to prepare for data analysis.^[17]^ The quality assessment of the articles was done by 2 trained experts. Each expert evaluated the quality of the entered articles separately. If at the end of the review there was a discrepancy between the 2 experts in terms of grading the articles, the third expert would make a decision in this regard.

## 3. Screening of studies

The retrieved articles were given to 2 trained people through EndNote version 8 software. At first, the title and abstract of the articles were reviewed and any that did not meet the inclusion criteria were removed by each of these individuals. In the next step, the articles were examined in terms of full text. The full text of the articles was reviewed, and if the desired data were provided, the data were entered into a predetermined checklist. Two authors (SH and RZ) independently performed the screening of studies, extraction of results, and evaluation of quality control of articles. In case there were any disagreements between the 2, the supervisor (HD) would make a final comment on the article. The most important reasons for removing the articles included the unavailability of full text, lack of entry criteria or exclusion criteria, unclear target population, failure to report the survival rate as a frequency (percentage).

### 3.1. Data extraction form

All final papers included in the research process were provided through a prewritten checklist with instructions for data extraction.

This checklist includes author name, publication year, study period (first year of period), country of origin, human development index (HDI), sample size, and annual survival rate for survival period.

### 3.2. Statistical analysis

The Cochran *Q* test, with a significant level of <0.1 has been used to assess the heterogeneity of studies using I2 statistical data. In terms of heterogeneity, a randomized effect model was used with the inverse algorithm. Methods such as meta-regression and subgroup analysis have been used in the event of heterogeneity between studies. Studies based on the survival rate of 5 years have been analyzed in the first stage. A meta-regression analysis was carried out at the other stage, due to a large heterogeneity of studies. The HDI was one of the indexes employed for this purpose. By year of study and sample size, meta-regression was also performed. For subgroup analysis, gender was considered. All analyses were fulfilled by comprehensive meta-analysis statistical software version 2.

### 3.3. Risk of bias

In studies, the Random Effect Model has been used to mitigate bias risks. In order to assess the risk of publication bias, a test was also applied by Egger.^[18]^ A funnel plot typically plots effect sizes against their standard errors or precisions. In the presence of diffusion bias, the funnel plot is expected to be skewed.^[19]^

### 3.4. Ethical consideration

As this study was a systematic review of previously published literature, ethical approval was not were not required.

## 4. Results

### 4.1. Research selection

**Figure 1.**
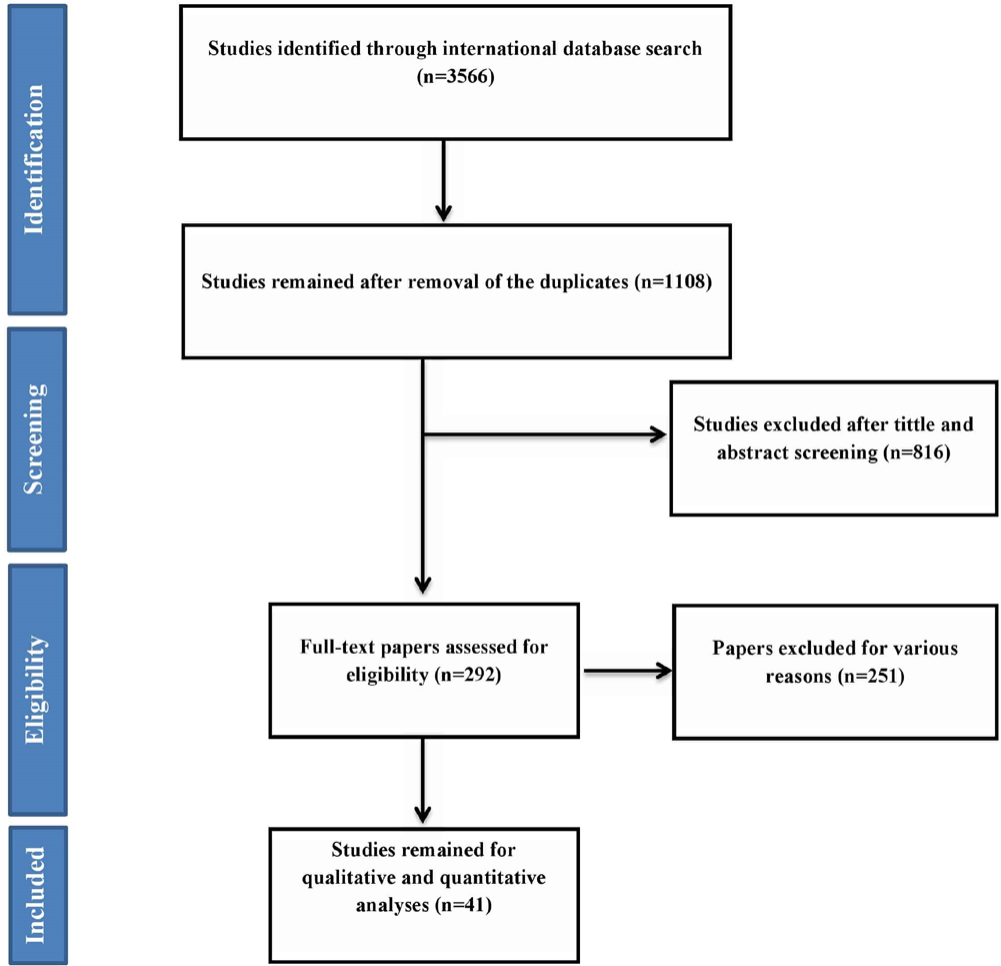
Study protocol.

Following the search of all international databases, 3566 articles were found and a total of 1108 citations were found after the removal of duplicate articles. Articles were chosen for full text 292 revision following a review of their titles and abstracts. Lastly, 41 papers were arrived for the meta-analysis. The reference lists of included papers were also evaluated to add related studies. Many papers were excluded for a variety of details, which included unrelated topics (N = 816), unrelated populations (N = 117), inadequate data (N = 129), and repeated finding (N = 5). Figure 1 provides an overview of the study selection process.

### 4.2. Study characteristics

From 1998 to 2022, the studies included have been made publicly available. According geographical locations, 14 studies were conducted in Korea,^[20–33]^ 14 in China,^[34–47]^ 6 in Japan,^[48–53]^ 1 in Singapore,^[54]^ 4 in Thailand,^[55–58]^ 1 in India^[59]^ and 1 in Sri Lanka.^[60]^ Table 1 illustrates the characteristics of the studies covered.

**Table 1:** Basic information of included studies.

Order	Author, year	Location	Time period	Sample size	Overall 5-yr survival rate (%)
1	Ahn, 2011	Korea	1993	1177	68.90
2	Ajiki, 2004	Japan	1975	122	71.31
3	Chen, 2011	China	1992	72	26.39
4	Chen, 2011	China	1982	59	37.29
5	Chia, 2011	Singapore	1993	369	50.68
6	Chia, 2011	Singapore	1988	244	42.62
7	Hong, 2020	Korea	1993	42,513	64.30
8	Hong, 2020	Korea	1996	42,513	67.00
9	Hong, 2020	Korea	2001	42,513	73.60
10	Hong, 2020	Korea	2006	42,513	78.60
11	Hong, 2020	Korea	2011	42,513	82.40
12	Hong, 2020	Korea	2013	42,513	83.10
13	Jiang, 2020	China	2004	276	90.58
14	Jung, 2011	Korea	2004	3228	76.39
15	Jung, 2012	Korea	2005	3435	77.09
16	Jung, 2014	Korea	2007	3989	78.79
17	Jung, 2015	Korea	2008	4152	79.89
18	Kang, 2022	Korea	2015	6026	84.70
19	Law, 2011	China	1996	914	66.19
20	Li, 2017	China	2000	401	60.35
21	Martin, 1998	Thailand	1983	114	15.79
22	Martin, 2011	Thailand	1990	46	34.78
23	Moon, 2014	Korea	1993	35	68.57
24	Moon, 2014	Korea	1996	94	76.60
25	Moon, 2014	Korea	2001	142	87.32
26	Moon, 2014	Korea	2006	209	87.08
27	Nakagawa-Senda, 2017	Japan	2006	1106	56.06
28	Oh, 2016	Korea	2008	4333	80.80
29	Pan, 2015	China	1998	142	83.10
30	Qu, 2018	China	2004	1299	73.98
31	Shao, 2020	China	1999	1202	87.60
32	Shin, 2011	Korea	1996	706	61.61
33	Sriplung, 2011	Thailand	1990	46	36.96
34	Tsukuma, 2006	Japan	1993	3637	63.98
35	Vatanasapt, 1998	Thailand	1985	70	22.86
36	Wang, 2018	China	2000	337	82.49
37	Wei, 2019	China	2004	1274	83.59
38	Woo, 2011	Korea	1997	279	67.03
39	Xiang, 2011	China	1992	743	53.30
40	Xishan, 2011	China	1991	1094	60.60
41	Yeole, 2011	India	1992	819	36.02
42	Zaitsu, 2020	Japan	2004	5265	71.98
43	Zaitsu, 2022	Japan	1992	704	86.51
44	Zeng, 2015	China	2003	2597	61.99
45	Zeng, 2018	China	2006	15,671	65.00
46	Zeng, 2018	China	2009	15,671	64.70
47	Zeng, 2018	China	2012	15,671	69.80
48	Zhou, 2021	China	2012	442	59.50

### 4.3. Quality appraisal

We found 36 studies with good quality, while 5 of them had a fair quality (Supplementary 2, Supplemental Digital Content, https://links.lww.com/MD/P662).

### 4.4. Results of the meta-analysis

In the first place, the articles were sorted according to the year of publication, and then the survival rate was analyzed over a period of 5 years. It is important to note that the number of papers on the 2 and 4 year survival rates was very low, and the results of the one, 3, and 10 year survival rates were not very satisfactory.

### 4.5. Overall 5-year survival rate

**Figure 2.**
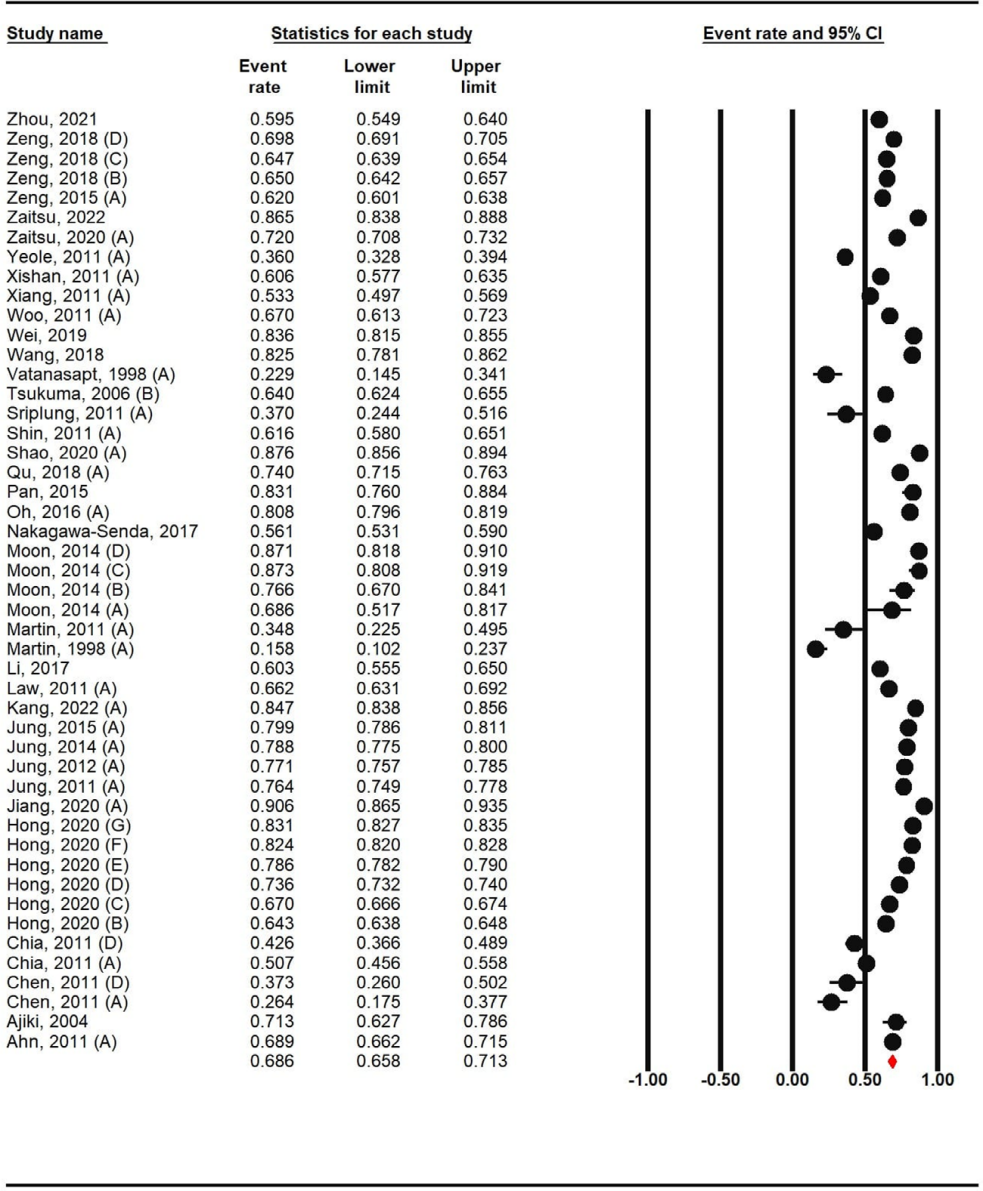
Overall 5-year survival rate of kidney cancer in Asia.

A survival rate of 68.60% was observed with a 95% confidence interval between 65.80% and 71.30 % over 5 years. Survival rates have been varied across time periods and various countries. The lowest overall 5-year survival rate was related to the study of Martin et al,^[56]^ who estimated the survival rate of this cancer in 1983 in Thailand to be about 15.79% with a 95% confidence interval of 10.20% to 23.70.3%. A another study, conducted in China by Chen et al,^[34]^ was able to determine a survival rate of 26.39% with a 95% confidence interval from 17.50% to 37.70%. However, the highest survival rate was also reported in Jiang’s study^[43]^ in China. Based on a 95% confidence interval of 86.50% to 93.50%, the 5 year survival rate in this study is estimated to be 90.58%. Shao et al study^[40]^ in China reported a survival rate of 87.60%. Another study by Moon et al^[32]^ in Korea reported a survival rate of about 87%. The general estimation of the survival rate in the conducted studies shows that the countries of Japan and Korea have the highest percentages, China has an average survival rate, and the countries of Thailand, Singapore and China have a lower survival than other studies (Fig. 2).

### 4.6. Five-year survival rate in male

**Figure 3.**
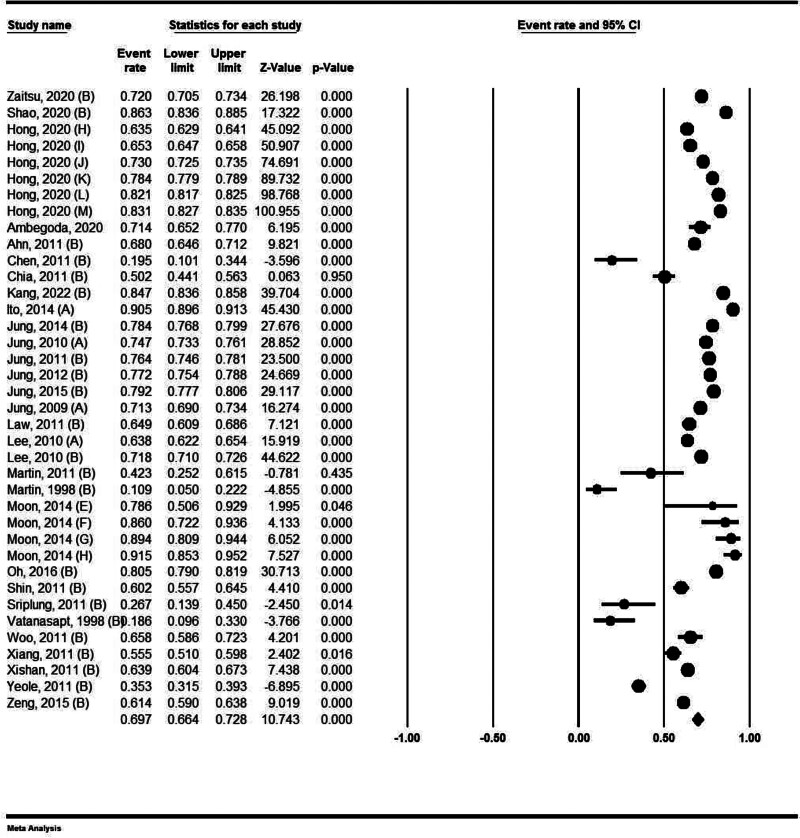
Five-year survival rate of kidney cancer in male.

The 5-year survival rate in men was 69.70%, with a 95% confidence interval of 66.40% to 73.80%. Martin et al reported a survival rate of <5 years for men in their study. The survival rate of this cancer in the period of 1983 in Thailand was estimated to be 10.90%. In a study by Moon et al in Korea, the highest survival rate has also been reported for men at 91.5% (Fig. 3).

### 4.7. Five-year survival rate in female

**Figure 4.**
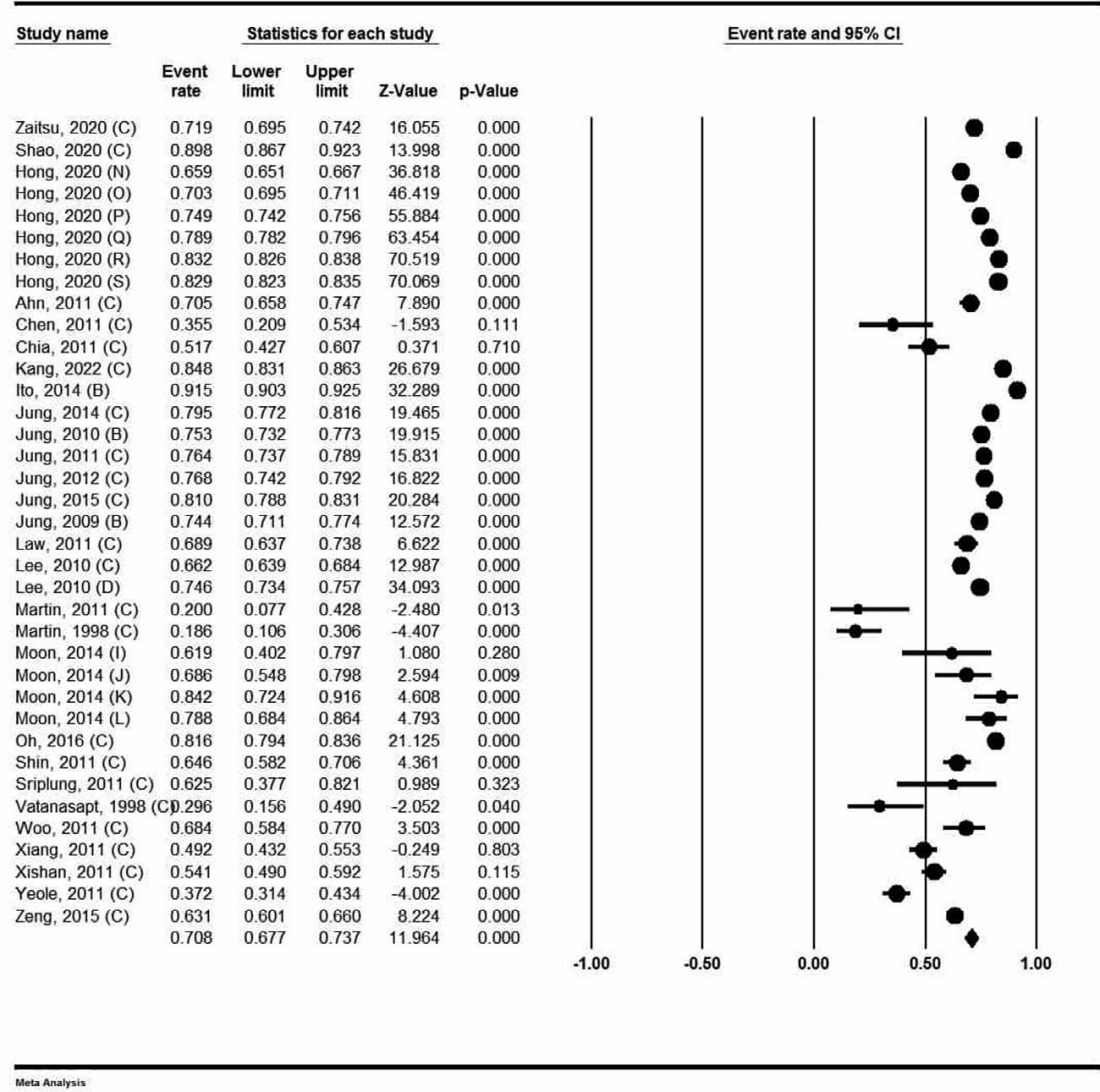
Five-year survival rate of kidney cancer in female.

In women, the 5-year survival rate was 70.80%, with a 95% confidence interval of 67.70% to 73.70%. Martin et al also reported the lowest survival rate of 5 years for females in 1983 data. The survival rate of RCC in Thailand is estimated to be 18.60% according to the results of this study. In the Ito et al study in Japan, the highest survival rate for women was reported at (91.5%).^[51]^ (Fig. 4).

### 4.8. Meta-regression

**Figure 5.**
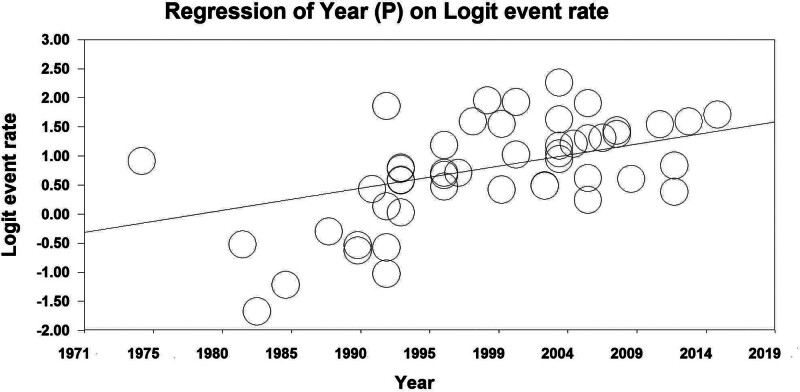
Results of meta-regression of the relationship between survival rate of kidney cancer and study time.

**Figure 6.**
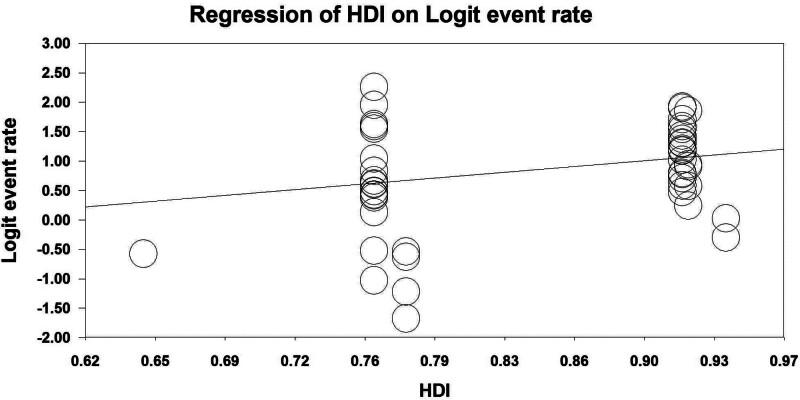
Results of meta-regression of the relationship between survival rate of kidney cancer and HDI. HDI = human development index.

A significant association between publication year and 5 years of survival has been shown by the results of a regression analysis. Thus, the year of study is a cause of variability in results of 5-year (Reg Coef = 0.039, *P* < .001) survival rates. In line with the results, an increased survival rate has been observed throughout the study period shown in Figure 5. HDI was another factor that contributed to inconsistent results. HDI was a cause of variability in results of 5-year survival rates (Reg Coef = 2.79, *P* < .001). In countries with higher HDI, an increased survival rate has been observed, as shown in Figure 6.

### 4.9. Risk of bias results

**Figure 7.**
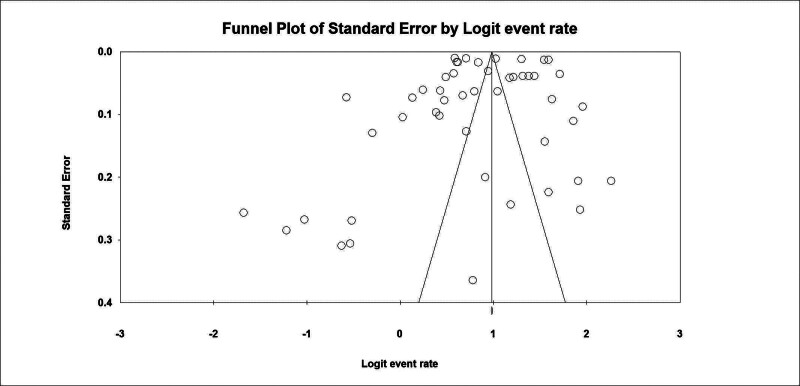
Funnel plot.

A *P*-value of .493 was obtained from the regression intercept test by Egger, which does not show any evidence of publication bias. Furthermore, no publication bias is detected on the funnel chart diagram of Figure 7.

## 5. Discussion

In the present study 5-year survival rate for RCC were examined, which were 68.60%. The 5-year survival rate of this cancer in men and women was also discussed, which was 69.7% and 70.8%, respectively. Kidney cancer, with a 5-year survival rate of 76%, from 2009 to 2015, is known as the deadliest urological cancer. The rate was 50 in the 1970s, rising to 57% in 1980 and 73% in 2000.^[61]^ In a similar study conducted in Canada between 2004 and 2008, the overall 5 year survival rate for kidney cancer was found to be 68 %, in line with the current findings.^[62]^ Meanwhile, the 5-year relative survival rate in US adults is reported to be 77.1%.^[63]^ Another study conducted in European countries showed that the 5-year relative survival for kidney cancer in Europe is about 60%; Austria and Germany had the highest survival rates at about 71 and 70.2%, respectively.^[64]^

A study by Pelant et al Between 1998 and 2009 in central and northern Denmark found that the overall 1-year survival of kidney cancer improved from 56% in 1998 to 63% in 2009. The same study also predicted that the 5-year survival rate would increase from 33 to 42 percent. In general, survival was increased in all age and sex groups (except men under 60).^[65]^ A study comparing the relative 5-year survival rates of kidney cancer in Japan, the United States, and Europe found that the 15- to 44-year-old age group showed a survival rate of about 70 percent for both sexes. The age group over 70 years showed a survival rate of about 40%. No gender differences were observed in survival. Men in Japan and women in Japan and the United States showed relatively high survival rates for all age groups.^[66]^ Another study by Tingru Miao et al In the United States found that the total 5-year survival of kidney cancer in men and women was 64.70% and 67.50%, respectively, which is lower than the findings of the current research. Also, this study showed that the 5-year survival rate of the Asian race is about 68.50%, which was significantly higher than the white race.^[67]^ Our research found that kidney cancer 5-year survival rates in Asian countries vary greatly, ranging from 15.8% to 90.6% depending on the type of cancer. However, it is important to note that these rates are not representative of the survival potential of all Asia. There are several factors that may contribute to differences in survival rates, including variations in healthcare systems, risk factors, genetics, and screening and early detection programs.^[12,68]^

There are some countries in Asia that have higher survival rates for kidney cancer. For example, a study conducted in Japan reported that the 5-year survival rate for kidney cancer was 86.5%, which is higher than the average survival rate in other Asian countries.^[53]^ This may be due to Japan’s advanced healthcare system, early detection programs, management patients and high availability of advanced treatments.^[69]^

On the other hand, a study conducted in India reported that the 5-year survival rate for kidney cancer was only 36%, which is lower than the average survival rate in other Asian countries.^[59]^ This may be attributed to the lack of access to advanced treatments and the high prevalence of risk factors such as smoking and obesity. The ease of access to health resources and the high quality of the infrastructure and resources of the health and treatment network can significantly affect the survival rate. Therefore, it is expected that countries with advanced healthcare systems have higher survival rates compared to other countries.^[2,70,71]^ Also, advanced countries in the field of health and treatment, which have strong screening programs and campaigns to inform people about patients, experience earlier detection (increasing the chance of incidental diagnosis of tumors) and, subsequently, higher survival rates.^[3,5]^ Areas with more people at low socio-economic levels may have lower survival due to reasons such as: higher prevalence of unhealthy behaviors such as smoking and alcohol consumption, poor diet, lack of health insurance and less access to medical and health care.^[72]^

Mortality and survival of patients with kidney cancer depend on 2 essential factors: patient age and tumor size. It is thought that people with tumors smaller than 5 cm had the highest survival rate. Patients over the age of 70 also had the highest mortality, regardless of tumor size. Surgery is the best therapeutic intervention compared to systemic treatments and radiation therapy. The 5-year survival rate after surgery is generally more effective in younger people with smaller tumors. One-third of older people die after successful surgery for reasons other than kidney cancer.^[73]^ Also, some studies have shown that marriage can be one of the issues affecting the survival of patients, so the survival rate of kidney cancer in married patients is better than single patients, and in the group of single people, widows are more at risk that psychological and physiological factors are influential in this regard.^[67]^ In general, the differences in renal cancer survival rates observed in study populations, time periods and sample sizes can be explained methodologically by a variety of factors.

A significant association between publication years and survival rates for 5 years has been identified by the results of this study. Therefore, variability in the 5 year survival rate is due to a period of study. In line with the results, a higher survival rate was observed during the study period. One of the main reasons for this has been to improve the quantitative and qualitative level of cancer diagnosis and to discover better treatments than in the past. A survey by Kaire Innos et al in Estonia from 1995 to 2014 showed that the relative 5-year survival rate improved from 54% in 1995 to 66% in 2014.^[74]^

The results show that in countries there is an increased survival rate due to the high HDI levels. Overall, the results showed that in Japan and Korea, with higher HDI, the 5 year survival rate was higher. On the other hand, survival rates have been lower in countries such as India and Thailand, which have relatively lower HDI. Because access to treatments and interventions is better provided in countries with higher HDI, patients in the initial phases of cancer are treated, and the probability of survival increases. Asia consists of several countries varying regarding their socio-economic properties and health care systems. Such differences can affect the survival rate of different diseases including cancers. Daroudi et al in an ecological study assessed the effect of different socio-economic factors including gross domestic product, healthcare expenditure, and life expectancy on the 5-year survival of common cancers. Their study revealed that gross domestic product and life expectancy are 2 factors that can significantly increase the survival rate of cancers like breast cancer.^[75]^ Exarchakou et al assessed the socio-economic inequalities’ effects on cancer survival by studying 1.2 million patients in England. They found that more disadvantaged patients, especially young individuals facing more deadly forms of cancer, experience a greater reduction in years of life compared to those who are less disadvantaged.^[76]^ A systematic review by Afshar et al discussed socio-economic variation’s impact on cancer survival. They stated that lower socio-economic status inversely affects the renal cancer survival rate.^[77]^ The difference in socio-economic status and health system power among various countries results in different medication availability, insurance support, screening programs, and health care services which all affect the survival rate of cancers.^[78,79]^ Another factor that affects the survival of kidney cancer in Asia is ethnic distribution. Previous studies showed the impact of ethnicity on cancer survival. Ellis et al reported that Blacks had lower cancer survival compared to Whites.^[80]^

The risk of harm caused by therapeutic interventions in patients with limited life expectancy is greater than the benefits that these patients receive. These patients are unable to treat cancers that progress slowly (such as kidney cancer in the early stages). These therapeutic interventions show their effect slowly and these people do not live long to see the benefits of the treatment. Also, these patients are at high risk of death during their lifetime due to causes other than cancer.^[81–84]^ The chosen treatment method can also depend on the level of life expectancy, for example, a person with a high life expectancy has more treatment choices than someone with a low life expectancy. Because these people are more focused and active on problems, and since they have high hopes, they tolerate long treatments and side effects of treatment methods better, and they follow treatment methods more. But people with lower life expectancy are less likely to seek expensive and costly treatment methods and invasive medical care, which practically eliminates a group of treatment methods. Therefore, with these interpretations, it seems that patients who have a longer life expectancy have better overall treatment results.^[85,86]^

### 5.1. Strengths

This study has several notable strengths. First, our meta-analysis incorporated data from 41 studies, yielding a substantial total sample size that enhances the statistical power and precision of the estimated 5-year survival rate in kidney cancer patients. Second, we utilized a random-effects model to account for potential heterogeneity across studies, ensuring that our findings robustly reflect variations in study populations and methodologies.

### 5.2. Study limitations

The lack of access to the full text of some articles was one of the limitations in this study. We were not provided with the complete text of this article, despite correspondence to its authors. Another limitation of the study was the impossibility of survival analysis based on cancer stage and type of treatment received. Also, the lack of examination and estimation of 1, 3, and 10-year survival and sensitivity analysis due to data limitations and the small number of studies is considered one of the limitations of the study.

## 6. Conclusion

In general, the total survival for kidney cancer in Asia is lower than in Western countries. This may be due to a lack of awareness about the disease and its symptoms, leading to delayed diagnosis and treatment. In addition, some Asian countries may have limited access to advanced medical technologies and treatments, which can impact survival rates. Countries with higher HDIs, such as Japan and South Korea, also had higher survival rates. Despite increased kidney cancer survival in recent years, early diagnosis and effective treatment and control programs are still necessary. Also, the educational program for the whole community, especially people at higher risk, can help in the primary diagnosis of the cancer and the more prolonged survival of cancer.

### 6.1. Practical suggestions for future research

The findings of this meta-analysis, while clarifying survival patterns in patients with kidney cancer, highlight several areas for future research. First, conducting meta-analyses based on individual-based data (IPD) could allow for a more detailed examination of subgroups based on clinical criteria such as tumor stage, treatment protocols, and molecular subtypes. Second, given the heterogeneity of reporting methods in existing studies, the development of standard guidelines for presenting survival results (including Kaplan–Meier curves with confidence intervals) seems necessary. Third, the gender-specific findings in this study, which indicate possible differences in outcomes, highlight the need to investigate biological mechanisms and the impact of gender on treatment response. From a geographical perspective, expanding future research to underrepresented regions (especially low-income countries) could provide a more comprehensive picture of global disparities in access to treatment. Finally, a more in-depth study of long-term survivors (>5 years) and the application of artificial intelligence methods to analyze heterogeneous datasets could open new horizons in understanding protective factors and nonlinear predictors of survival. These research directions not only cover the limitations of the present study, but also, building on the findings of this analysis, outline the next steps towards improving the clinical management of this disease.

## Acknowledgments

The present study is a student research project (No. 1400-101) conducted at Larestan University of Medical Sciences. The present study was approved by the ethics code IR.LARUMS.REC.1400.016. The authors would like to thank the Student Research Committee of Larestan University of Medical Sciences for supporting this project.

## Author contributions

**Conceptualization:** Hamed Delam, Zahra Keshtkaran.

**Data curation:** Hamed Delam, Reza Zare, Ahmadreza Eidi, Mohammad Sadegh Moradi Sarcheshmeh, Alireza Shahedi, Meghdad Abdollahpour-Alitappeh.

**Formal analysis:** Hamed Delam, Soheil Hassanipour.

**Investigation:** Hamed Delam, Reza Zare.

**Methodology:** Hamed Delam.

**Project administration:** Hamed Delam.

**Software:** Hamed Delam.

**Supervision:** Hamed Delam.

**Validation:** Hamed Delam, Mohammad Sadegh Moradi Sarcheshmeh.

**Writing – original draft:** Hamed Delam, Reza Zare.

**Writing – review & editing:** Hamed Delam, Reza Zare, Zahra Keshtkaran, Ehsan Amini-Salehi.

## Supplementary Material


