# Supplementary figures and images for: Five-year survival rate of kidney cancer (localized renal cell carcinoma) in the Asia: A systematic review and meta-analysis
Source: Medicine (Baltimore). 2025 Aug 22;104(34):e43867. doi: 10.1097/MD.0000000000043867 (PMC12384827; doi:10.1097/MD.0000000000043867)

**Search strategy**

PubMed/Medline


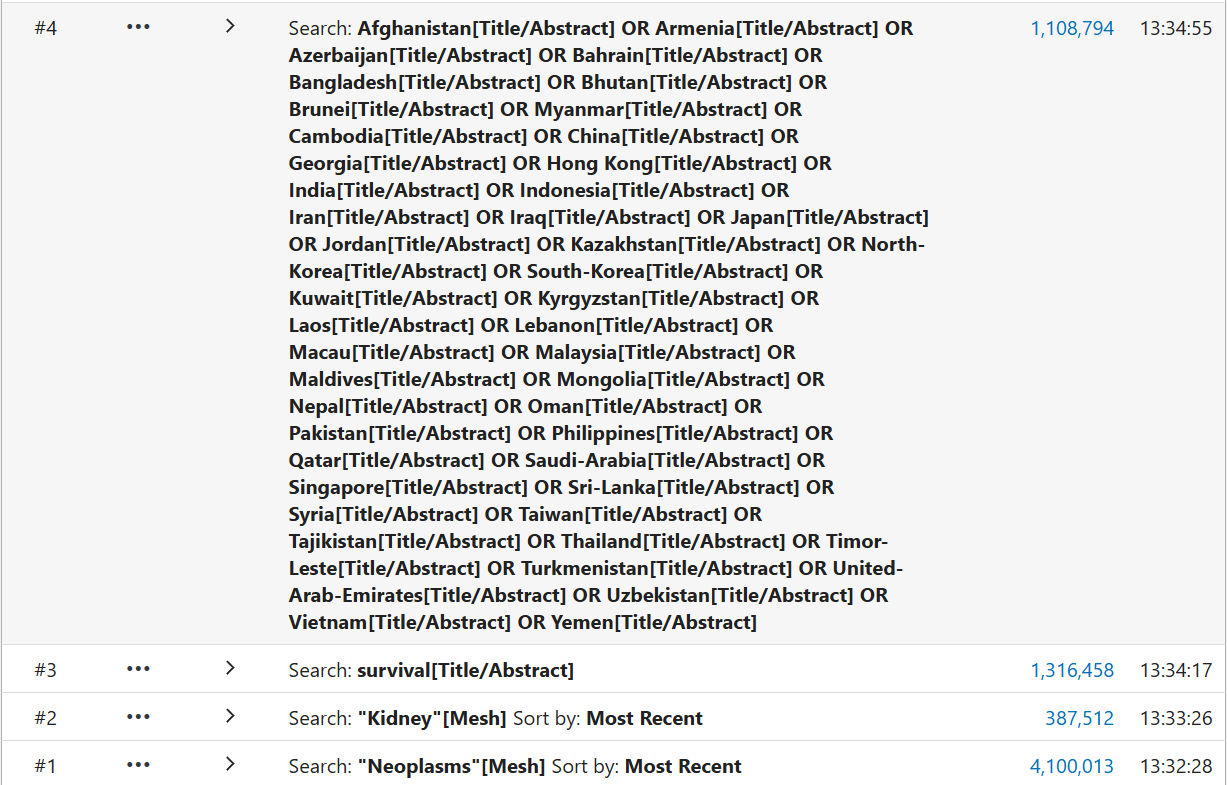

Supplement: Supplementary file 1 [file medi-104-e43867-s001.docx]
