# Supplementary material for: Five-year survival rate of kidney cancer (localized renal cell carcinoma) in the Asia: A systematic review and meta-analysis
Source: Medicine (Baltimore). 2025 Aug 22;104(34):e43867. doi: 10.1097/MD.0000000000043867 (PMC12384827; doi:10.1097/MD.0000000000043867)
Supplement: Supplementary file 2 [file medi-104-e43867-s002.docx]

**Newcastle-Ottawa Quality Assessment Form**

| **Author (year)** | **Selection** | **Comparability** | **Exposure/Outcome** | **Total** | **Quality** |
| --- | --- | --- | --- | --- | --- |
| Ahn, 2011 | 3 | 1 | 2 | 6 | Good |
| Ajiki, 2004 | 2 | 1 | 2 | 5 | Fair |
| Chen, 2011 | 3 | 1 | 2 | 6 | Good |
| Chia, 2011 | 3 | 1 | 3 | 7 | Good |
| Hong, 2020 | 2 | 1 | 3 | 6 | Good |
| Jiang, 2020 | 3 | 1 | 3 | 7 | Good |
| Jung, 2011 | 3 | 1 | 3 | 7 | Good |
| Jung, 2012 | 3 | 1 | 2 | 6 | Good |
| Jung, 2014 | 3 | 1 | 3 | 7 | Good |
| Jung, 2015 | 3 | 1 | 3 | 7 | Good |
| Kang, 2022 | 3 | 1 | 3 | 7 | Good |
| Law, 2011 | 3 | 1 | 2 | 6 | Good |
| Li, 2017 | 3 | 1 | 3 | 7 | Good |
| Martin, 1998 | 2 | 1 | 2 | 5 | Fair |
| Martin, 2011 | 3 | 1 | 2 | 6 | Good |
| Moon, 2014 | 3 | 1 | 3 | 7 | Good |
| Nakagawa-Senda, 2017 | 3 | 1 | 3 | 7 | Good |
| Oh, 2016 | 3 | 1 | 3 | 7 | Good |
| Pan, 2015 | 3 | 1 | 2 | 6 | Good |
| Qu, 2018 | 3 | 1 | 3 | 7 | Good |
| Shao, 2020 | 3 | 1 | 2 | 6 | Good |
| Shin, 2011 | 3 | 1 | 2 | 6 | Good |
| Sriplung, 2011 | 3 | 1 | 2 | 6 | Good |
| Tsukuma, 2006 | 2 | 1 | 2 | 5 | Fair |
| Vatanasapt, 1998 | 3 | 1 | 2 | 6 | Good |
| Wang, 2018 | 3 | 1 | 3 | 7 | Good |
| Wei, 2019 | 3 | 1 | 2 | 6 | Good |
| Woo, 2011 | 3 | 1 | 3 | 7 | Good |
| Xiang, 2011 | 3 | 1 | 3 | 7 | Good |
| Xishan, 2011 | 3 | 1 | 2 | 6 | Good |
| Yeole, 2011 | 3 | 1 | 2 | 6 | Good |
| Zaitsu, 2020 | 3 | 1 | 2 | 6 | Good |
| Zaitsu, 2022 | 3 | 1 | 3 | 7 | Good |
| Zeng, 2015 | 3 | 1 | 3 | 7 | Good |
| Zeng, 2018 | 3 | 1 | 3 | 7 | Good |
| Zhou, 2021 | 3 | 1 | 3 | 7 | Good |
| Lee, 2010 | 3 | 1 | 3 | 7 | Good |
